# Comparison of porcine milk microRNA expression in milk exosomes versus whole swine milk and prediction of target genes

**DOI:** 10.5194/aab-65-37-2022

**Published:** 2022-01-26

**Authors:** Zhihong Liu, Yuchun Xie, Juntao Guo, Xin Su, Cun Zhao, Chongyan Zhang, Qing Qin, Dongliang Dai, Yanan Tuo, Zongyuan Li, Danni Wu, Jinquan Li

**Affiliations:** 1 College of Animal Science, Inner Mongolia Agricultural University, Hohhot 010018, Inner Mongolia, China; 2 Key Laboratory of Animal Genetics, Breeding and Reproduction, Hohhot 010018, Inner Mongolia Autonomous Region, China; 3 Key Laboratory of Mutton Sheep Genetics and Breeding, Ministry of Agriculture, Hohhot 010018, China; 4 Engineering Research Center for Goat Genetics and Breeding, Hohhot 010018, Inner Mongolia Autonomous Region, China

## Abstract

Milk exosomal microRNAs (miRNAs) are important for postnatal growth and immune system maturation in newborn mammals. The functional hypothesis of milk exosomal miRNAs and their potential bioavailability in milk to newborn mammals were investigated. Briefly, 37 exosomal miRNAs were upregulated compared to miRNAs found outside the exosomes. Among these miRNAs, ssc-miR-193a-3p expression was upregulated 1467.35 times, while ssc-miR-423-5p, ssc-miR-551a, ssc-miR-138, ssc-miR-1 and ssc-miR-124a were highly concentrated and upregulated 13.58–30.06 times. Moreover, these miRNAs appeared to be relevant for cell development and basic physiological processes of the immune system. Following the analysis of target gene prediction and related signalling pathways, 9262 target genes were mainly concentrated in three signalling pathways: metabolic pathways, pathways in cancer, and phosphatidylinositol 3-kinase/protein kinase B (PI3K/Akt) signalling pathways. Among 9262 target genes, more than 20 miRNAs were enriched in exosomes, such as methyl CpG binding protein 2 (MECP2) and glycogen synthase 1 (GYS1). After determining the miRNA localization-, distribution- and function-related metabolism, we found that these exosomes were specifically concentrated miRNA target genes and they were interrelated with cell development and basic cell functions, such as metabolism and immunity. It is speculated that miRNAs in milk can influence offspring via milk exosomes.

## 1 Introduction

Milk is an important source of nutrition and a vehicle of passive immunity for infants. It contains numerous immune components indispensable for infant brain development and growth. Additionally, it is a rich source of miRNAs that are essential for neonatal innate and adaptive immunity. There are mutually exclusive hypotheses regarding the function of mother's milk: (1) that the miRNAs in milk can be passed to the newborn and thus are relevant for physiologic adjustment and (2) that milk merely provides nutritional benefits (Melnik et al., 2016).

Chen et al. (2010) detected 245 different kinds of miRNAs in cow's milk. Moreover, using Illumina's Solexa Sequencing Technology, it has been confirmed that 213 different kinds of miRNAs are present in ordinary cow milk (Chen et al., 2010). RNAs of bovine milk exosomes have been detected in tissues (including the brain) of mice after oral administration of milk exosomes (Manca et al., 2018). Furthermore, miRNAs in animal body fluids have often been shown to be selectively wrapped in exosomes. Kosaka et al. (2010) found that miRNA molecules are stable even in very acidic conditions, indicating that milk allows for the dietary intake of miRNAs by infants. miRNAs present in milk were able to enter normal and tumour cells and affect their biological functions. For example, milk exosome-derived miRNA-148a via DNMT1 suppression reduces the methylation status of CpG islands of promoters of crucial developmental genes (Golan-Gerstl et al., 2017).

Exosomes contain a number of proteins and fats, as well as a number of nucleotides (mRNA and microRNA) (Liu et al., 2018). Exosomes have an important role in transporting RNAs to target cells. RNAs in high-content exosomes are relevant for the delivery of genetic information between tissues and low-content exosomes, e.g. tumour and immune cell-derived exosomes, have been shown to carry tumour antigens and promote immunity, leading to the eradication of established tumours by CD8 + T cells and CD4 + T cells, as well as directly suppressing tumour growth and improving resistance to malignant tumour development (David et al., 2015). Furthermore, miRNAs in exosomes can resist RNase degradation and be preserved in serum and blood plasma, and they can be used as tags for clinical and biological diagnosis (Lin et al., 2016). Zhang et al. (2014) proposed that exosomes and their products could serve as valid gene and drug delivery carriers in cancer therapy. For example, higher miR-92a-3p and miR-17-5p expression in circulating exosomes was clearly associated with the pathological stages and grades of colorectal cancer (Fu et al., 2017). miRNAs in exosomes may have an important role as delivery carriers and biological tags. We further analysed and discussed the content and function of miRNAs in swine milk exosomes.

## 2 Materials and methods

### 2.1 Ethics statement

All methods used in this study were performed in accordance with protocols approved by the Laboratory Animal Management Committee of the Chongqing Academy of Animal Sciences and the Ministry of Science and Technology of the Peoples Republic of China (approval number: 2006398).

### 2.2 Animals

Fresh swine milk samples were collected from the pig breeding farm of the Chongqing Academy of Animal Science, China. Three individuals were randomly selected for biological analysis replicates. After 31 d of lactation, we removed the piglets and collected swine milk, which was quickly frozen in liquid nitrogen and stored for analysis.

### 2.3 Exosomal extraction

Exosomes were isolated with the ExoQuick-TC (System Biosciences, USA) according to the manufacturer's instructions. Briefly, biofluids were collected from 10 mL milk and centrifuged at 3000×g for 15 min to remove cells and cell debris. Two millilitres (mL) of ExoQuick-TC Exosome Precipitation Solution was added to the supernatants, mixed by vortexing and stored at 4 ${}^{\circ }$ C overnight. Then, the mixture was centrifuged at 1500×g for 30 min at room temperature, the supernatant was discarded, and the pellet was resuspended in 100–500 µL of 1X RIPA buffer. Finally, the exosomes were extracted and observed by scanning electron microscopy.

### 2.4 RNA extraction

RNA extraction was conducted as follows: 10 mL of sample was mixed with 2 mL ExoQuick-TC, placed at 4 ${}^{\circ }$ C overnight (6 h), and centrifuged at 1500×g for 30 min, and the supernatant was discarded. The exosome pellet was resuspended in 350 µL LYSIS buffer, vortexed for 15 s, and placed at room temperature for 5 min. The solution was then mixed with 200 µL of 100 % ethanol, vortexed for an additional 10 s, transferred to a fresh spin column, and centrifuged at 13 000 rpm for 1 min. The flow-through was discarded, and the spin column was placed back into a collection tube, mixed with 400 µL of WASH buffer and centrifuged at 13 000 rpm for 1 min. The flow-through was discarded, and the solution was centrifuged at 13 000 rpm for 2 min to dry. Subsequently, the collection tube was discarded, while the spin column was assembled with a fresh, RNase-free 1.5 mL elution tube. ELUTION buffer (30 µL ) was directly added to the membrane in a spin column and centrifuged at 2000 rpm for 2 min. The speed was increased to 13 000 rpm and it was centrifuged for 1 min.

To control the quality of miRNA and cDNA synthesis, a miRCURY LNA™ Universal RT microRNA PCR RNA Spike-in kit (Exiqon) was used. The total RNA quantity and purity were analysed by a Bioanalyser 2100 and RNA 6000 Nano Lab Chip Kit (Agilent, CA, USA) with RIN number >7.0 . To control the quality of the RNA extraction and cDNA synthesis for microRNA qPCR, a miRCURY LNA™ Universal RT microRNA PCR RNA Spike-in kit (Exiqon) was used.

**Figure 1:**
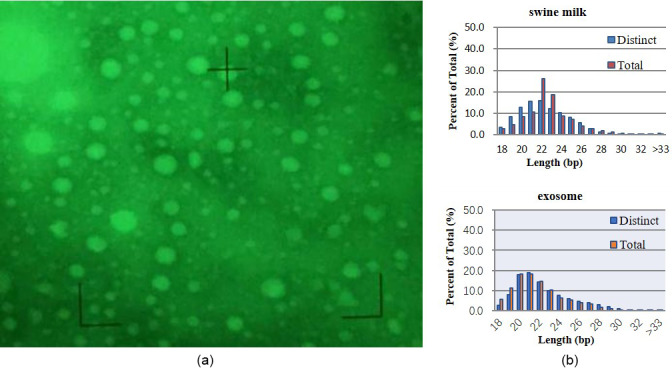
Basic analysis. **(a)** Exosomes isolated from swine milk. **(b)** Statistics of miRNA length from different samples.

### 2.5 Small RNA library construction and sequencing

Approximately 1 µg of total RNA was used to prepare a small RNA library according to the protocol of TruSeq Small RNA Sample Prep Kits (Illumina, San Diego, USA). We performed single-end sequencing (36 bp) on an Illumina HiSeq 2500 at LC-BIO (Hangzhou, China) following the vendor's recommended protocol.

The original data were initially removed from the 3 ${}^{\prime }$ adapter sequence, while sequences shorter than 18 nt in length were discarded. If there were 80 % A, G, C, or T in the sequence, only A or C with no G or T, or only G or T with no A, C, or continuous nucleotide dimers or trimers, these sequences were filtered out. By comparing the measured sequences with the mRNA, rRNA, tRNA, snRNA, and Repbase databases, clean reads were obtained.

**Figure 2:**
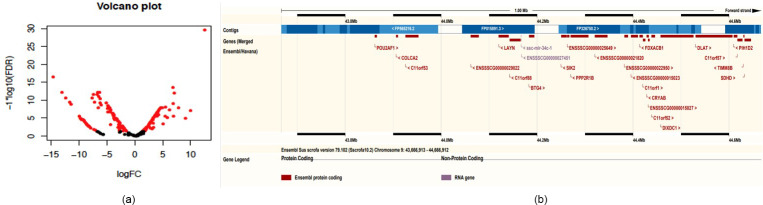
Bioinformatics analysis of microRNA. **(a)** Volcano plot of differential miRNAs between exosomes and milk (red indicates differentially expressed miRNAs). **(b)** Mapping of the miRNA cluster on chromosome 9.

**Figure 3:**
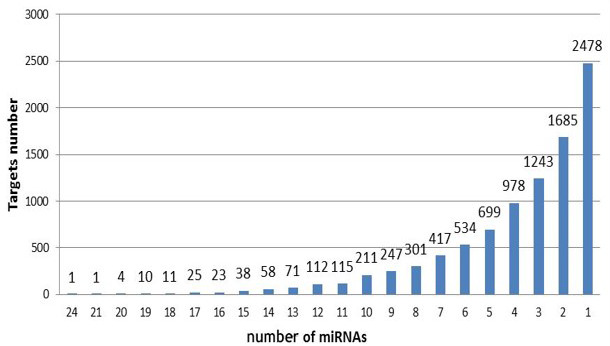
The number of target genes corresponding to miRNA.

### 2.6 Conservative analysis of miRNA

The remaining reads of ncRNAs such as rRNA and tRNA were removed (miRbase 20) to identify and obtain miRNA expression levels: if the mature miRNA was in the [ x , y ] region of the pre-miRNA and the sequencing quantity was in the position [ x-2 , y+5 ], it was counted as the expression amount of the mature miRNA.

### 2.7 miRNA difference between exosome and swine milk

EdgeR was used to analyse the differences between the miRNAs: a P value <0.05 was considered statistically significant, a ratio >2 times higher was considered different, and a ratio >10 times higher was considered significantly different.

### 2.8 Upregulated miRNA in exosome

*Family analysis and gene cluster analysis.* According to the position of pre-miRNA in the genome, we treated the miRNA within the range (10 kb) as a miRNA cluster. miRNAs were classified by a family of 2 to 8 bp seed regions of the mature body. The family name corresponding to the seed sequence was classified as a reference to TargetScan (http://www.targetscan.org/, last access: 25 December 2014). Prediction of miRNA target genes with miRNAs was performed using the default parameter of free energy <-20 $\mathrm{kcal}\;{\mathrm{mol}}^{-1}$ . Thirty-seven upregulated miRNAs in exosomes were analysed according to http://www.kegg.jp/kegg/pathway.html (last access: 27 December 2014) to map pathways.

## 3 Results

### 3.1 Ultrastructural features of porcine milk exosomes

Electron microscopy revealed exosomes that are represented by small vesicles of different sizes (diameter of 40–100 nm) (Fig. 1a). The samples were of good shape, high density, low impurity, and the expected size.

### 3.2 Subtractive libraries

By sequencing swine milk samples (R-1, R-2, R-3) and exosome samples (E-1, E-2, E-3), we obtained 10 088 489, 10 955 079, 9 907 545, 10 785 934, 14 208 994, and 16 651 629 clean reads. microRNAs with 18–34 bases were found in milk and exosomes, and their length was consistent with a normal distribution (approximately 22 nt) (Fig. 1b).

### 3.3 Conservative analysis of microRNA

By searching the swine miRNA data in miRbase 20.0 and performing bow-tie comparison, we obtained 280 pre-miRNAs and 326 mature miRNAs (Table 1). Furthermore, 236 pre-miRNAs and 243 conserved miRNAs were found in swine milk, and 208 pre-miRNAs and 200 mature miRNAs were found in exosomes.

**Table 1:** Statistical analysis of microRNAs.

Class	Pre-miRNA	Mature	Unique sRNA mapped	Total sRNA mapped
			pre-miRNA	pre-miRNA
Known microRNAs	280	326	–	–
in miRbase				
Swine milk	236	243	2838	1 794 033
Exosome	208	200	1613	234 133

### 3.4 miRNA content of swine milk and swine exosomes

Compared to miRNAs found in swine milk, 2-fold higher expression was observed for 37 exosomal miRNAs and 10-fold higher expression was observed for six exosomal miRNAs (Table 2, Fig. 2a). Among these, the level of ssc-miR-193a-3p expression was 1467.35 times higher in exosomes than in milk. Five additional exosomal miRNAs, i.e. ssc-miR-423-5p, ssc-miR-551a, ssc-miR-138, ssc-miR-1, and ssc-miR-124a, were found to be concentrated and upregulated (13.58 to 30.06-fold higher expression) in exosomes.

**Table 2:** High concentration of miRNA in exosomes.

	Database				Exosome vs. swine milk			
micoRNA	Sequence	Length	Swine milk	Exosome	P value	Ratio	Mark	Mark
							2 times	10 times
ssc-miR-193a-3p	AACTGGCCTACAAA	22	75	67 154	$1.33\times 10^{-32}$	1467.35	UP	UP
	GTCCCAGT							
ssc-miR-423-5p	TGAGGGGCAGAG	23	1302	22 588	$4.23\times 10^{-16}$	30.06	UP	UP
	AGCGAGACTTT							
ssc-miR-551a	GCGACCCACTCTTG	20	11	225	$3.75\times 10^{-14}$	25.43	UP	UP
	GTTTCC							
ssc-miR-138	AGCTGGTGTTGTGA	21	1	42	$1.90\times 10^{-9}$	13.95	UP	UP
	ATCAGGC							
ssc-miR-1	TGGAATGTAAAGAA	21	3	58	$8.12\times 10^{-11}$	13.94	UP	UP
	GTATGTA							
ssc-miR-124a	TAAGGCACGCGGTG	21	0	33	$1.83\times 10^{-8}$	13.58	UP	UP
	AATGCCA							

### 3.5 Analysing 37 upregulated miRNAs detected in swine milk and exosomes

*Family analysis and gene cluster analysis.* There were 41 pre-miRNAs obtained belonging to 30 families (Table 3). Two mature miRNAs were found in miR-146, miR-17, miR-221, and miR-30, while all of the others had a single mature miRNA.

**Table 3:** Family analysis of upregulated miRNAs in exosomes.

Number	Mature microRNA	Pre-miRNAs	Family
1	ssc-miR-2	ssc-mir-1	mir-1
2	ssc-miR-124a	ssc-mir-124a-2	mir-124
3	ssc-miR-124a	ssc-mir-124a-1	mir-124
4	ssc-miR-1272	ssc-mir-1271	mir-1271
5	ssc-miR-130a	ssc-mir-129a	mir-129
6	ssc-miR-139	ssc-mir-138	mir-138
7	ssc-miR-140-4p	ssc-mir-140	mir-140
8	ssc-miR-146a-6p	ssc-mir-146a	mir-146
9	ssc-miR-147b	ssc-mir-146b	mir-146
10	ssc-miR-107a	ssc-mir-106a	mir-17
11	ssc-miR-17-4p	ssc-mir-17	mir-17
12	ssc-miR-181d-6p	ssc-mir-181d	mir-181
13	ssc-miR-186	ssc-mir-185	mir-185
14	ssc-miR-193a-4p	ssc-mir-193a	mir-193
15	ssc-miR-205	ssc-mir-205	mir-205
16	ssc-miR-210	ssc-mir-210	mir-210
17	ssc-miR-219	ssc-mir-219	mir-219
18	ssc-miR-221-4p	ssc-mir-221	mir-221
19	ssc-miR-223	ssc-mir-222	mir-221
20	ssc-miR-92b-6p	ssc-mir-92b	mir-25
21	ssc-miR-28a	ssc-mir-27a	mir-27
22	ssc-miR-296-4p	ssc-mir-296	mir-296
23	ssc-miR-30a-4p	ssc-mir-30a	mir-30
24	ssc-miR-30c-4p	ssc-mir-30c-2	mir-30
25	ssc-miR-321	ssc-mir-320	mir-320
26	ssc-miR-331-4p	ssc-mir-331	mir-331
27	ssc-miR-339-4p	ssc-mir-339-1	mir-339
28	ssc-miR-34c	ssc-mir-34c-1	mir-34
29	ssc-miR-34c	ssc-mir-34c-2	mir-34
30	ssc-miR-345-3p	ssc-mir-345-1	mir-345
31	ssc-miR-345-3p	ssc-mir-345-2	mir-345
32	ssc-miR-365-6p	ssc-mir-365-2	mir-365
33	ssc-miR-378	ssc-mir-378-1	mir-378
34	ssc-miR-378	ssc-mir-378-2	mir-378
35	ssc-miR-423-6p	ssc-mir-423	mir-423
36	ssc-miR-455-3p	ssc-mir-455	mir-455
37	ssc-miR-455-5p	ssc-mir-455	mir-455
38	ssc-miR-552a	ssc-mir-551a	mir-551
39	ssc-miR-4332	ssc-mir-4331	
40	ssc-miR-4334-4p	ssc-mir-4334	
41	ssc-miR-7134-6p	ssc-mir-7134	

By chromosome localization of 41 upregulated pre-miRNAs in exosomes and 5 M selection, 9ch and Xch had gene clusters (Table 4). ssc-mir-34c-1 and ssc-mir-34c-2 were located in 44166873_44166952 on 9ch and fixed on 45274873_45274942 and 45275613_45275692 on Xch. Two miRNAs on 9ch are members of the miR-34 family, and the miRNAs are located on many upstream genes between the LAYN and SIK2 genes. In addition, there were new miRNAs at this site (Fig. 2b). Two miRNAs on Xch are members of the mir-221 family. There are new miRNAs in the 10 k range where no gene exists.

**Table 4:** Statistics table of upregulated miRNA clusters in exosomes.

Cluster	Number of pre-miRNA	Pre-miRNAs	
Cluster 1	2	ssc-mir-34c-1 (9_44166873_44166952_-)	ssc-mir-34c-2 (9_44166877_44166950_-)
Cluster 2	2	ssc-mir-221 (X_45274873_45274942_-)	ssc-mir-222 (X_45275613_45275692_-)

**Table 5:** Top 10 target genes corresponding to miRNAs.

Targets	Gene	Description	Number of miRNAs	miRNA
ENSSSCT 00000035620	MECP2	methyl CpG binding protein 2 (Rett syn- drome)	24	ssc-miR-1271 ∣ ssc-miR-138 ∣ ssc-miR-140-3p ∣ ssc-miR-146a-5p ∣ ssc-miR-17-3p ∣ ssc-miR-185 ∣ ssc-miR-205 ∣ ssc-miR-219 ∣ ssc-miR-222 ∣ ssc-miR-296-3p ∣ ssc-miR-30c-3p ∣ ssc-miR-320 ∣ ssc-miR-331-3p ∣ ssc-miR-339-3p ∣ ssc-miR-345-3p ∣ ssc-miR-34c ∣ ssc-miR-365-5p ∣ ssc-miR-378 ∣ ssc-miR-423-5p ∣ ssc-miR-4331 ∣ ssc-miR-4334-3p ∣ ssc-miR-455-5p ∣ ssc-miR-7134-5p ∣ ssc-miR-92b-5p
ENSSSCT 00000003502	GYS1	Sus scrofa glycogen synthase 1 (muscle) (GYS1), mRNA	21	ssc-miR-106a ∣ ssc-miR-140-3p ∣ ssc-miR-146a-5p ∣ ssc-miR-17-3p ∣ ssc-miR-185 ∣ ssc-miR-193a-3p ∣ ssc-miR-210 ∣ ssc-miR-296-3p ∣ ssc-miR-30c-3p ∣ ssc-miR-320 ∣ ssc-miR-331-3p ∣ ssc-miR-339-3p ∣ ssc-miR-34c ∣ ssc-miR-365-5p ∣ ssc-miR-378 ∣ ssc-miR-423-5p ∣ ssc-miR-4331 ∣ ssc-miR-4334-3p ∣ ssc-miR-551a ∣ ssc-miR-7134-5p ∣ ssc-miR-92b-5p
ENSSSCT 00000006227	LRRC8A	leucine rich repeat containing 8 family, member A	20	ssc-miR-1271 ∣ ssc-miR-138 ∣ ssc-miR-140-3p ∣ ssc-miR-146a-5p ∣ ssc-miR-185 ∣ ssc-miR-193a-3p ∣ ssc-miR-219 ∣ ssc-miR-27a ∣ ssc-miR-30c-3p ∣ ssc-miR-331-3p ∣ ssc-miR-339-3p ∣ ssc-miR-345-3p ∣ ssc-miR-34c ∣ ssc-miR-365-5p ∣ ssc-miR-378 ∣ ssc-miR-4331 ∣ ssc-miR-4334-3p ∣ ssc-miR-455-3p ∣ ssc-miR-7134-5p ∣ ssc-miR-92b-5p
ENSSSCT 00000016465	BCL9L	B-cell CLL/lymphoma 9-like	20	ssc-miR-1271 ∣ ssc-miR-138 ∣ ssc-miR-17-3p ∣ ssc-miR-185 ∣ ssc-miR-205 ∣ ssc-miR-210 ∣ ssc-miR-296-3p ∣ ssc-miR-30a-3p ∣ ssc-miR-30c-3p ∣ ssc-miR-320 ∣ ssc-miR-331-3p ∣ ssc-miR-339-3p ∣ ssc-miR-345-3p ∣ ssc-miR-34c ∣ ssc-miR-365-5p ∣ ssc-miR-423-5p ∣ ssc-miR-4331 ∣ ssc-miR-455-3p ∣ ssc-miR-551a ∣ ssc-miR-92b-5p
ENSSSCT 00000024857	PHRF1	PHD and ring finger domains 1	20	ssc-miR-1271 ∣ ssc-miR-129a ∣ ssc-miR-138 ∣ ssc-miR-140-3p ∣ ssc-miR-181d-5p ∣ ssc-miR-219 ∣ ssc-miR-221-3p ∣ ssc-miR-296-3p ∣ ssc-miR-30c-3p ∣ ssc-miR-331-3p ∣ ssc-miR-339-3p ∣ ssc-miR-345-3p ∣ ssc-miR-365-5p ∣ ssc-miR-378 ∣ ssc-miR-423-5p ∣ ssc-miR-4331 ∣ ssc-miR-4334-3p ∣ ssc-miR-455-3p ∣ ssc-miR-7134-5p ∣ ssc-miR-92b-5p
ENSSSCT 00000030861	ELOVL1	ELOVL fatty acid elongase 1	20	ssc-miR-1 ∣ ssc-miR-106a ∣ ssc-miR-1271 ∣ ssc-miR-140-3p ∣ ssc-miR-17-3p ∣ ssc-miR-219 ∣ ssc-miR-221-3p ∣ ssc-miR-222 ∣ ssc-miR-27a ∣ ssc-miR-296-3p ∣ ssc-miR-30c-3p ∣ ssc-miR-331-3p ∣ ssc-miR-339-3p ∣ ssc-miR-345-3p ∣ ssc-miR-34c ∣ ssc-miR-378 ∣ ssc-miR-423-5p ∣ ssc-miR-4331 ∣ ssc-miR-455-5p ∣ ssc-miR-92b-5p
ENSSSCT 00000000472		uncharacterized protein	19	ssc-miR-129a ∣ ssc-miR-140-3p ∣ ssc-miR-17-3p ∣ ssc-miR-185 ∣ ssc-miR-205 ∣ ssc-miR-219 ∣ ssc-miR-221-3p ∣ ssc-miR-222 ∣ ssc-miR-296-3p ∣ ssc-miR-30c-3p ∣ ssc-miR-331-3p ∣ ssc-miR-339-3p ∣ ssc-miR-34c ∣ ssc-miR-365-5p ∣ ssc-miR-378 ∣ ssc-miR-423-5p ∣ ssc-miR-4331 ∣ ssc-miR-4334-3p ∣ ssc-miR-7134-5p
ENSSSCT 00000002656	VASH1	vasohibin 1	19	ssc-miR-1271 ∣ ssc-miR-129a ∣ ssc-miR-138 ∣ ssc-miR-140-3p ∣ ssc-miR-193a-3p ∣ ssc-miR-219 ∣ ssc-miR-222 ∣ ssc-miR-27a ∣ ssc-miR-296-3p ∣ ssc-miR-30a-3p ∣ ssc-miR-320 ∣ ssc-miR-331-3p ∣ ssc-miR-339-3p ∣ ssc-miR-34c ∣ ssc-miR-365-5p ∣ ssc-miR-4331 ∣ ssc-miR-4334-3p ∣ ssc-miR-455-3p ∣ ssc-miR-455-5p
ENSSSCT 00000005027	ZNF609	zinc finger protein 609	19	ssc-miR-106a ∣ ssc-miR-124a ∣ ssc-miR-129a ∣ ssc-miR-138 ∣ ssc-miR-140-3p ∣ ssc-miR-146a-5p ∣ ssc-miR-181d-5p ∣ ssc-miR-185 ∣ ssc-miR-205 ∣ ssc-miR-296-3p ∣ ssc-miR-30c-3p ∣ ssc-miR-320 ∣ ssc-miR-331-3p ∣ ssc-miR-339-3p ∣ ssc-miR-34c ∣ ssc-miR-365-5p ∣ ssc-miR-378 ∣ ssc-miR-423-5p ∣ ssc-miR-92b-5p
ENSSSCT 00000011944	NAV1	uncharacterized protein	19	ssc-miR-106a ∣ ssc-miR-1271 ∣ ssc-miR-129a ∣ ssc-miR-138 ∣ ssc-miR-140-3p ∣ ssc-miR-17-3p ∣ ssc-miR-185 ∣ ssc-miR-296-3p ∣ ssc-miR-30c-3p ∣ ssc-miR-320 ∣ ssc-miR-331-3p ∣ ssc-miR-34c ∣ ssc-miR-365-5p ∣ ssc-miR-378 ∣ ssc-miR-423-5p ∣ ssc-miR-4331 ∣ ssc-miR-4334-3p ∣ ssc-miR-455-3p ∣ ssc-miR-92b-5p

*Target gene analysis*. With target gene prediction of 37 upregulated miRNAs in exosomes, we obtained 9262 target genes and their following miRNAs (Fig. 3). Six target genes corresponded to 20 miRNAs, 674 to more than 10 miRNAs, 8582 to less than 10 miRNAs, and 2478 to a single miRNA.

There were 6 target genes that corresponded to 20 miRNAs (Table 5). Methyl CpG binding protein 2 (MECP2) had the highest number of corresponding target genes, Sus scrofa glycogen synthase 1 (muscle) (GYS1), leucine rich repeat containing 8 family, member A (LRRC8A), B-cell CLL/lymphoma 9-like (BCL9L), PHD and ring finger domains 1 (PHRF1), ELOVL fatty acid elongase 1 (ELOVL1). Among 37 miRNAs, 24 miRNAs are thought to regulate MECP2, which may play a role in transcriptional regulation of neurons by directly regulating DGCR8/Drosha compounds and consequently influence the production of miRNA and expression of target genes (Chen, 2010; Wang et al., 2014; Woo and Kim, 2014).

**Figure 4:**
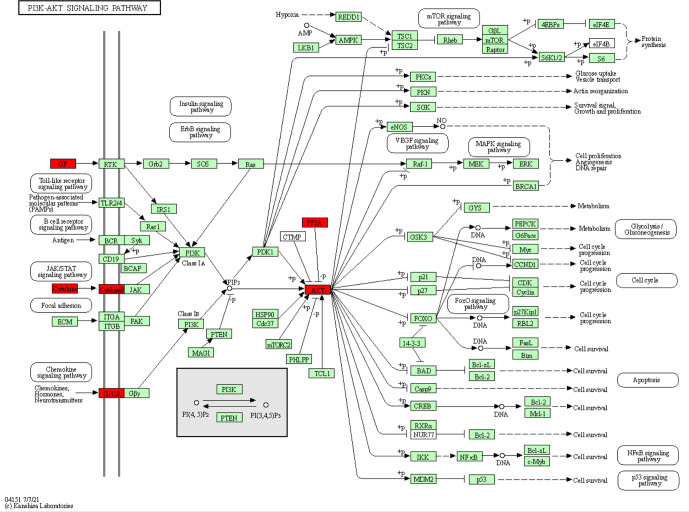
The location of the upregulated miRNA target genes in the PI3K-Akt signalling pathway. The target genes of the upregulated miRNA are red.

*Exosome signalling pathways analysis*. Using KEGG analysis with the target prediction for 37 upregulated miRNAs in exosome, 9262 target genes were obtained, mainly concentrated on three signalling pathways, i.e. metabolic, cancer, and PI3K-Akt signalling pathways (Fig. 4). Three signalling pathways maintained a relationship with immunity and growth.

## 4 Discussion

We selected upregulated miRNAs in milk, performed basic sequencing, and examined the differential expression profile of miRNAs between exosomes and swine milk. We found that some miRNAs were selectively over-enriched by exosomes. Swine milk exosomes have been previously found to affect immunity by increasing the concentration of miRNAs (Gu et al., 2012). We found six upregulated miRNAs: ssc-miR-423-5p, miR-193a, ssc-miR-551a, ssc-miR-138, ssc-miR-1, and ssc-miR-124a. Urinary exosomal miR-193a has been used as a biomarker for the diagnosis of primary focal segmental glomerulosclerosis in children (Huang et al., 2017; Khoo et al., 2017). Moreover, miR-193a-3p has been associated with tumour proliferation, transportation, and apoptosis (Yi et al., 2016). Five additional exosomal miRNAs, ssc-miR-423-5p, ssc-miR-551a, ssc-miR-138, ssc-miR-1, and ssc-miR-124a, were found to be concentrated and upregulated (13.58 to 30.06-fold higher expression) in the exosomes, which are involved in the upregulation of important signalling molecules, such as p-AKT and p-ERK1/2. In clinical samples, miR-423-5p is dysregulated, and a corresponding alteration in ING-4 expression has been observed. Upregulation of miR-551a inhibits cell proliferation and induces apoptosis in demethoxycurcumin-hindered ovarian cancer (Du et al., 2017). miR-1 upregulates the expression of SFRP1 in bladder cancer cells; excessive miR-1-3p's 3p expression can stimulate the immune attack of bladder cancer cells, inhibiting their proliferation and movement (Shang et al., 2017). miR-124a can enhance SIRT1 and induce CD4 + T cells to differentiate Tregs, illustrating ways of influencing neuropathic pain inflammation (Heyn et al., 2016). In summary, these findings suggest that the miRNAs play a role in basic physiological processes of cell development and immunity.

There were 6 target genes that corresponded to 20 miRNAs, MECP2, GYS1 BCL9L, PHRF1, and ELOVL1 (Table 5). Since most of the abundant miRNAs in exosomes can regulate MECP2, this may pave the way for the study of the effect of miRNAs in milk exosomes on the development of infant brains. Milk exosome miRNA-mediated suppression of DNMT1 and MECP2 may thus synergistically modify transcription and opening of chromatin structure, a critical epigenetic switch for growth and anabolism during the postnatal period (Melnik and Schmitz, 2017). GYS1 is interrelated with glycogen metabolism, and its mutation influences muscle glycogenosis (Baird et al., 2010; Fang et al., 2014). LRRC8A gene codes are involved in different cell biological processes, including cell adhesion, transportation, and receptor–hormone interactions, and they play an important role in B cell development, whose defects result from B cell maturation deflection (Okada et al., 2016; Platt et al., 2017). BCL9L is an essential gene of growth and immunity. Lack of this gene can enhance chromosome endurance and regulate the proliferation of heteroploids through the Wnt signalling pathway (Roche et al., 2008; Sannino et al., 2016). PHRF1 affects the transportation into the nucleus of the cancer inhibitory factor cPML, blocking the signal transduction of TGF- β /Smad. ELOVL1 has activity in fatty-acid-extended enzymes, impacting the metabolism of ω3 and ω6 , which are related to some diseases, such as brain degeneration (López-García et al., 2017). Using chromosome localization and target gene analyses, we discovered that these genes were related to development, fat acid metabolism, and immunity.

Furthermore, we found that the PI3K-Akt signalling pathway stimulated by various cells can regulate basic cellular functions, such as transcription, proliferation, translation, and development (Asati et al., 2016). PI3K is the second messenger that activates Akt, while PIP3 is catalysed by the cell membrane. Consequently, Akt could be relevant for functions such as apoptosis, generation of protein, metabolism, and cell cycle (Sizek et al., 2019). The finding that milk-derived miRNAs that promote the PI3K-AKT pathway are abundantly expressed in exosomes supports the functional role of milk exosomal miRNAs in mammalian physiology, as previously suggested (Ettahar et al., 2013; Wang et al., 2018). Highly expressed miRNAs and their target genes participate in basic cell functions and processes such as development, metabolism, and immunity.

## 5 Conclusions

After performing in-depth research on miRNA localization, distribution, and function-related metabolism, we found that these exosome-concentrated miRNA target genes were interrelated with development and basic cell functions, such as metabolism and immunity. It is speculated that miRNAs in milk can influence offspring via milk exosomes.

## Data Availability

All data files are available from the SRA database () as of 1 October 2024, accession number: PRJNA760432.SRA, submission ID: SUB10315543.
